# Transmission Modes of Melioidosis in Taiwan

**DOI:** 10.3390/tropicalmed3010026

**Published:** 2018-02-28

**Authors:** Pei-Tan Hsueh, Wei-Tien Huang, Hsu-Kai Hsueh, Ya-Lei Chen, Yao-Shen Chen

**Affiliations:** 1 Department of Internal Medicine, Kaohsiung Veterans General Hospital, Kaohsiung 813, Taiwan; mulberrymonster@gmail.com; 2 Department of Biotechnology, National Kaohsiung Normal University, Kaohsiung 824, Taiwan; milo.huang1121@gmail.com (W.-T.H); cuprousion@yahoo.com.tw (H.-K.H.)

**Keywords:** melioidosis, *Burkholderia pseudomallei*, transmission modes

## Abstract

In Taiwan, melioidosis is an emerging disease that suddenly increased in the Er-Ren River Basin, beginning in 2005 and in the Zoynan region during 2008–2012, following a typhoon. Additionally, the disease sporadically increased in a geography-dependent manner in 2016. Subcutaneous inoculation, ingestion, and the inhalation of soil or water contaminated with *Burkholderia pseudomallei* are recognized as the transmission modes of melioidosis. The appearance of environmental *B. pseudomallei* positivity in northern, central and southern Taiwan is associated with disease prevalence (cases/population: 0.03/100,000 in the northern region, 0.29/100,000 in the central region and 1.98/100,000 in the southern region). However, melioidosis-clustered areas are confined to 5 to 7.5 km^2^ hot spots containing high-density populations, but *B. pseudomallei*-contaminated environments are located >5 km northwestern of the periphery of these hot spots. The observation that the concentration of *B. pseudomallei*-specific DNA in aerosols was positively correlated with the incidence of melioidosis and the appearance of a northwesterly wind in a hot spot indicated that airborne transmission had occurred in Taiwan. Moreover, the isolation rate in the superficial layers of a contaminated crop field in the northwest was correlated with PCR positivity in aerosols collected from the southeast over a two-year period. The genotype ST58 was identified by multilocus sequence typing in human and aerosol isolates. The genotype ST1001 has increased in prevalence but has been sporadically distributed elsewhere since 2016. These data indicate the transmission modes and environmental foci that support the dissemination of melioidosis are changing in Taiwan.

## 1. Introduction

Melioidosis is an emerging and fatally infectious disease that is endemic to Southeast Asia and northern Australia [1]. However, its incidence may be underestimated in many regions, such as India, South America, and Africa, because the disease is difficult to diagnose due to its very diverse clinical manifestations and the use of inadequate bacterial isolation and identification methods. Annual disease rates could be increasing worldwide as a result of transmission by travelers that were exposed to contaminants in melioidosis-endemic areas [2,3,4]. Overall, approximately 165,000 human melioidosis cases occur annually worldwide, and approximately 89,000 people die annually from this disease [5]. To date, no systematic international estimation of the clinical or sub-clinical incidence of melioidosis has been performed using a consistent, standardized, and quality-controlled method under the same health- or medically-controlled conditions. This makes it difficult to adequately compare the incidence of the disease among countries or regions and to predict the disseminated scope of melioidosis.

The pathogen underlying melioidosis is an environmental saprophyte and Gram-negative bacterium called *Burkholderia pseudomallei* that is usually found in the soil and surface water, and is sometimes seen in air particles [6,7]. People acquire melioidosis from directly contacting, ingesting or inhaling soil, water or dust contaminated with *B. pseudomallei* [8,9,10]. Nevertheless, melioidosis cases are usually clustered during the rainy season, particularly after the appearance of a heavy rainfall, cyclone, or typhoon [11,12,13]. The transmission modes of melioidosis probably differ because people’s customs and lifestyles, as well as the geographical position of melioidosis case clusters differ. In this review, we focus on studies of the incidence of melioidosis in Taiwan that provide insight into the mechanism of melioidosis transmission.

## 2. Underestimated Period of Melioidosis in Taiwan (1984–2004)

The first reported case of melioidosis in Taiwan involved a patient who acquired melioidosis with multilobar pneumonia by drowning in the Philippines in 1984 [14]. Between 1984 and 2000, 12 pulmonary and two subcutaneous melioidosis cases and one melioidosis case with a mycotic aneurysm were reported [15]. Of those patients, 73.3% (11/15) had no overseas travel history. Because the symptoms of melioidosis are protean, melioidosis can go undetected. Its prevalence may therefore have been underestimated by physicians in Taiwan during the earlier years of its presence. Because *B. pseudomallei* is an uncertain airborne disease that belongs to a group of ‘selected agents’ that could be utilized to develop bacterial weapons for bioterrorism [16], melioidosis in Taiwan has been classified as a notifiable disease since 2000. This means that all of the culture-confirmed cases of melioidosis should be reported to the Centers for Disease Control (CDC) at the Department of Health in Taiwan. No case was reported from 2000 to 2002, five were reported in 2003, and 13 were reported in 2004. However, *B. pseudomallei* has consistently been isolated from soil at a depth of 30–60 cm in paddies in Taiwan and other countries [17,18]. In synthetic soil or water media, *B. pseudomallei* can survive for 150 days or more, indicating that affected Taiwanese individuals may have acquired melioidosis from indigenous bacteria, rather than by traveling to endemic areas [19]. To explore the potential for exposure to *B. pseudomallei* in Taiwan, an indirect ELISA (enzyme-linked immunosorbent assay) that targeted the truncated flagellin of the bacterium was developed [20]. Seropositive rates of approximately 4%, 2.8%, and 5% were detected in northern, central, and southern Taiwan, respectively [21]. Approximately 3% of the positive cases were patients with diabetes mellitus, which is a risk factor for melioidosis [22].

## 3. An Outbreak of Melioidosis in Taiwan (2005)

In 2005, after a typhoon event, melioidosis cases were clustered in the Er-Ren River Basin of Taiwan during a three-month period [23]. In 2005, while 0.3 cases/100,000 population were infected throughout the entire population of Taiwan, the incidence of melioidosis reached 122 cases/100,000 population in the area downstream of the Er-Ren River Basin. In the affected area, the incidence rates were higher than the 7.98–21.3 cases/100,000 population and 5.4–24.2 cases/100,000 population in Thailand and northern Australia, respectively [24,25,26]. Approximately 75% of these melioidosis patients had *B. pseudomallei* bacteremia, 50% had concomitant pleuropulmonary infections, and 20% died during hospitalization [27]. Pulsed-field gel electrophoresis (PFGE) typing revealed that two distinct genotypic clones appeared during this outbreak [27]. These two distinct types have been demonstrated to be ST58 and ST99 by the multilocus sequencing typing (MLST) method [28].

When compared with the seropositive rates of anti-*B. pseudomallei* flagellin antibodies of 2.8–5% in Taiwanese individuals before 2005, the rates were increased to as high as 36.6%, 21.6%, and 10.9% in the downstream, midstream, and upstream regions of the Er-Ren River, respectively. Those seropositive rates were associated with the incidence of melioidosis as follows: 120 cases/100,000 population in the downstream region, 68 cases/100,000 population in the midstream region and 36 cases/100,000 population in the upstream regions. However, *B. pseudomallei* was only isolated from the soil in the upstream region and never from the mid- or downstream regions, although PCR positivity to *B. pseudomallei*-specific amplicons was relatively higher in mid- and downstream regions than in upstream regions. People seemed to have acquired melioidosis through exposure to contaminated soil that had flowed down from the upstream river due to the heavy rain that was associated with the typhoon [26]. However, 70% (28/40) of the melioidosis patients in this outbreak denied recent contact with mud or dirty water before their illness [27]. Approximately 32.8–34.8% of the 105 adults who lived in the mid- and downstream areas of the Er-Ren River acknowledged having been flooded during this typhoon event [26].

After this outbreak, melioidosis was widely recognized as an indigenously emerging and fatally infectious disease in Taiwan. The CDC (Taiwan) reinforced the education and management of melioidosis using medical resources and improved the regulation of notifiable melioidosis in Taiwan. Thus, the numbers of melioidosis cases reported in the CDC database since this issue arose are more reliable than they previously were [29]. From 2005 to 2007, the average incidences of melioidosis were 0.03 cases/100,000 population in northern Taiwan, 0.29 cases/100,000 in central Taiwan and 1.98 cases/100,000 in southern Taiwan. The disease incidence was associated with PCR positivity for *B. pseudomallei*-specific amplicons, which was found in 1.3%, 10.1% and 19.4% of environmental samples in northern, central, and southern Taiwan, respectively. Viable *B. pseudomallei* was cultivated only from the soil in southern Taiwan (south of the Tropic of Cancer; isolation rate, 12.6%), and environmental isolates were never found in central and northern Taiwan (north of the Tropic of Cancer) [30]. *B. pseudomallei* is usually isolated from moist soil or water in tropical and subtropical countries, and is rarely found in temperate countries [1]. Although the favorable physical (pH and water content) and chemical (nutrients, salinity, organic, and inorganic compounds) conditions for the growth of *B. pseudomallei* are inconsistent in many soil studies [19,31,32,33,34], *B. pseudomallei* can thrive in rice fields when nutrients (e.g., fertilizer) are depleted during agriculture practices and rice field management [31]. After harvesting rice, the straw is frequently burned in the rice field in Taiwan. The presence or absence of *B. pseudomallei* related to rice field management in Taiwan is unknown. However, the soil, even if relatively dry, may act as a reservoir during the dry season, with an increase in the proliferation and potential for mobilization from soil into water in the wet season [35].

## 4. A Plateau in the Incidence of Melioidosis in Taiwan (2008–2012)

In 2008–2012, the annual incidence of melioidosis among the entire Taiwanese population increased to 0.19 cases/100,000 population as compared with 0.07 cases/100,000 population in 2007 [36]. Most of the cases were geographically clustered in the Er-Ren River Basin (size, 108 km^2^; population, 79,790 people) and Zoynan region (size, 60 km^2^; population, 127,438 people) in Taiwan [36,37]. The average incidences of melioidosis in both regions reached 16.7 cases/100,000 population (Er-Ren River Basin) and 10.9 cases/100,000 population, respectively, from 2008 to 2011 (Zoynan region). Approximately 62.5–67% of melioidosis patients were unemployed and had no recent travel history because most patients were elderly or had chronic diseases, such as diabetes, renal failure, or cancer [37,38]. Acute pulmonary melioidosis predominated (>70%). Furthermore, 88% of melioidosis cases were found during the rainy season, and 22% of the patients died [38]. Based on molecular tracing observations, ST99 (multilocus sequencing typing) was prevalent in the Er-Ren River Basin in 2005, but ST58 was found in the Zoynan region in 2008–2009, indicating that the epidemic strains of melioidosis were altered because the environmental foci contaminated with *B. pseudomallei* differed [28].

Two hot spots for melioidosis occurred 10.3 km apart, but the climatic conditions, including rainfall and wind strength, were different because the topographical characteristics differed, as described below.

The Er-Ren River Basin is a plain, and the Zoynan Region is a hilly area. Rainfall that lasts for a period of 4–7 days is a trigger factor associated with an increase in melioidosis cases in both areas. However, rainfall lasting 6–8 days and wind with a speed >19 m/s (>17.2 m/s is defined as a typhoon) and a specific angle of wind direction (150°, 220°, or 280°) are associated with increases in the number of melioidosis cases in the flat Er-Ren River Basin. In the hilly Zoynan region, these wind speeds, when combined with rainfall lasing five days, was also associated the incidence of melioidosis [37]. In Taiwan, over 80% of melioidosis cases occurred in the rainy season when the typhoon appeared [13,26,27]. It is not surprising that the wind speed (>19 m/s) was associated with the occurrence of melioidosis in Taiwan. When considering the epidemiological survey described above, in the Er-Ren River Basin, *B. pseudomallei* was isolated from upstream areas, but melioidosis cases were increased in the mid- and downstream areas [26]. The direction of the sampled site contaminated with *B. pseudomallei* at the hot spot is from the southeast to northwest. Thus, the wind, with a 150° angle, as a vector could assist the transmission of *B. pseudomallei* to the patients if the airborne particles (aerosols) were generated from contaminated soil or water.

In contrast, in the Zoynan region, if a strong northwest wind originating from a counterclockwise cyclone occurred during the season the typhoon appeared, the incidence of melioidosis should be higher in the southeast districts, even though *B. pseudomallei* is isolated from environments in the northwestern region. In particular, the hills located south of the Zoynan region become a natural barrier to obstruct the transmission of aerosols contaminated with *B. pseudomallei*. Indeed, 96% (80/83) of melioidosis cases occurred north of the hilly Zoynan region, but rare cases occurred south of the Zoynan region [37]. To test this hypothesis, we first demonstrated that the concentration of *B. pseudomallei*-specific amplicons was increased in the rainy season when the typhoon appeared when compared with that in the dry season when the appearance of a typhoon is rare [39]. By sampling randomly but with an even distribution geographically as much as possible, several sites contaminated with *B. pseudomallei* in the northwestern wetted, cropped, or non-cropped soil of the hot spots were identified. The concentration of *B. pseudomallei*-specific amplicons in aerosols was positively correlated with the incidence of melioidosis and the appearance of a northwesterly wind. Moreover, the isolation rate in the superficial layers of the contaminated cropped field in the northwest was correlated with PCR positivity for aerosols that were collected from the southeast from 2011–2013. The genotype ST58 of *B. pseudomallei* was identified from soil, aerosol, and human isolates. The airborne transmission of melioidosis was demonstrated to move from contaminated soil to aerosols and/or to humans in the Zoynan region [6].

## 5. A New Hot Spot of Melioidosis in Taiwan (At the Present Time)

**Figure 1:**
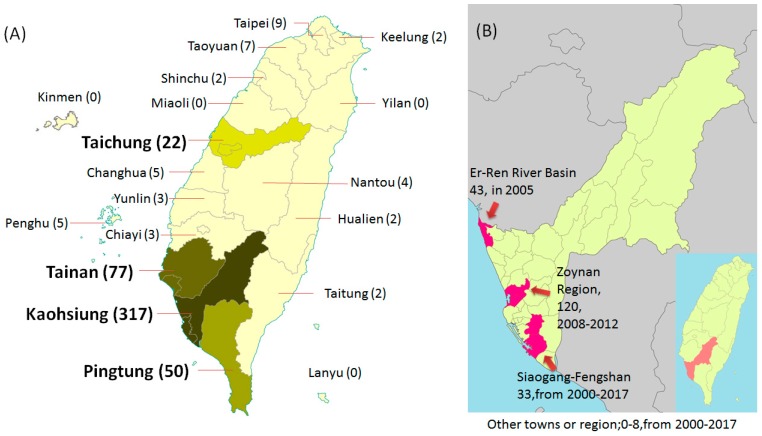
Geographical distribution of melioidosis in Taiwan. (**A**) In the entire island with the case number shown in brackets. (**B**) In Kaohsiung district. Two hot spots (Er-Ren River Basin and Zoynan Region) and Siagogan-Fengshan (location, case number, and year in which it occurred) are shown as red color.

From 2000 to 2017, 516 melioidosis cases were reported in Taiwan. As melioidosis is a tropical disease, approximately 86% (444/516) of cases are geographically distributed south of the Tropic of Cancer in Taiwan (Figure 1A). Over 50% (223/444) of the cases were successively clustered in the Er-Ren River Basin in 2005 and in the Zoynan region in 2008–2012, and in Siaogang–Fengshan, more sporadic cases with subcutaneously localized infection have occurred since 2003 (Figure 1B). Usually, acute pulmonary melioidosis with bacteremia is clustered after extreme climate events, for example, heavy rainfall and typhoons, but melioidosis with localized infection can occur in dry seasons. For example, patients who were admitted with melioidosis 7–14 days after heavy monsoonal rainfall (188 mm) were more ill or were more likely to die (rainfall >211 mm) in Australia [40]. Over 1000 mm of rainfall during the wet season and a wind speed of >19 m/s (Er-Ren River Basin) were indicators for melioidosis occurrence in Taiwan [27,37,38]. The genotype ST1001 of *B. pseudomallei* was found in melioidosis patients dwelling in Siaogang–Fengshan in 2016. ST58 was prevalent in soil and human isolates during 2008–2012, while ST1001 has been sporadically found in humans and the environment. Both representative strains, vgh07 (ST58) and vgh16 (ST1001), have been sequenced. The vgh07 (ST58) strain contains 16 genomic islands, including a potential pathogenic island that harbors an invasive hemolytic gene, but vgh16 (ST1001) did not harbor any genomic island [41,42]. When the BALB/c mice were infected with the strains intravenously and the strains were re-isolated from liver extracts and used to re-infect the mice intravenously, repeatedly, the phenotypic levels of 3-hydroxytetradecanoic acid (an indicator of lipopolysaccharide of *B. pseudomallei*), biofilm formation and flagellar expression in both types were significantly increased when compared with those in the original parent strains. Moreover, the survival rates of the mice infected with both types that re-isolated from liver extracts were obviously decreased, indicating that the animals, as reservoirs, could increase the virulence of *B. pseudomallei* [43]. An environmental strain isolated from Pahang, Malaysia, was reported to show up-regulation of adhesins, virulence factors, and stress response-related proteins when the strain was injected intra-peritoneally into a mouse and re-isolated from the spleen [44]. If ST1001 persists in animals that serve as reservoirs for a long time, this strain can develop an increased virulence.

More research is needed to explore the molecular links among the environment, animals, and humans in Siaogang-Fengshan. However, since 2015, political considerations have resulted in a CDC (Taiwan) policy that involves tightly regulating the biosecurity and biosafety of *B. pseudomallei*. The ability to use a large panel of *B. pseudomallei* strains for molecular typing, as was previously possible, is now restricted. Only six strains, including ST1001, were identified in the soil (*n* = 3), animals (*n* = 1), and humans (*n* = 2) in Siaogang-Fengshan from 2015 to 2017. Their virulence in animals has not yet been determined.

## 6. Unresolved Problems Related to Melioidosis Dissemination in Taiwan

Melioidosis in Taiwan has successively clustered in certain areas, such as the Er-Ren River Basin, Zoynan regions, and Siaogang-Fengshan, consisting of small-sized regions with areas containing approximately 5–7.5 km^2^, and has spread through different transmission modes or via exposure to different environmental foci [36]. As our insight into the geographical distribution of melioidosis cases and environmental foci improves, physicians will be able to increase alertness and awareness, leading to better control of the disease.

However, the pH value, solar strength, moisture, organic or inorganic content, temperature changes, and salinity of soil each affect the growth of *B. pseudomallei* in the environment [9,19,31,32,33,34]. To detect the presence of *B. pseudomallei* in the environment, the PCR detection rates are usually higher than those of microbiological cultures in our studies [6,26,30,36]. The presence of phages in *B. pseudomallei* strains has been reported to decrease their successful isolation from environmental samples [45]. Both *B. cenocepacia* and *B. multivorans*, which are antagonistic to *B. pseudomallei*, are widely distributed throughout the rice fields of Taiwan [46]. The secondary metabolites of *Bacillus amyloliquefaciens* isolated from soil can kill *B. pseudomallei* in vitro [47]. The amoeba *Paravahlkampfia ustiana* and some *Acanthamoeba* sp. are predators that graze on *B. pseudomallei*, while *Hartmannella* spp. and *Acanthamoeba astronyxis* may be reservoirs of *B. pseudomallei* in soil or water [48,49,50]. The physical or chemical parameters influencing the growth of *B. pseudomallei* in the environment are not completely understood in Taiwan. Although the geographical distribution of *B. pseudomallei* in the environment is very uneven, high isolation rates in soil or water are usually associated with a high incidence of melioidosis in surveys using a large area [26,30,36].

However, melioidosis-clustered cases are distributed within 5–7.5 km^2^ of the hot spot regions with high-density populations, but an environment contaminated with *B. pseudomallei* was located >5 km away from the periphery of the hot spot [6,36]. When most of the patients in a population are elderly or have diabetes and cannot participate in outdoor activities, a characterization of the vectors that carry contaminated *B. pseudomallei* should be performed [37,38]. The airborne transmission of infectious agents from aerosols to humans has been epidemiologically demonstrated using molecular linking and statistical methods. However, the finding that the isolation rates of *B. pseudomallei* in aerosols are very low contradicts the notion that the contaminated sources are contained in aerosols [6]. Additionally, in a rice paddy field in Ubon Ratchathani, northeast Thailand, *B. pseudomallei* was never isolated from air and rainwater samples during a 13-month prospective study [7]. In the absence of improved isolation techniques, it is uncertain whether environmental uncultivated *B. pseudomallei* can be considered an infective source.

Alternatively, it remains unclear how people become easily exposed to contaminated water or soil. In Taiwan, approximately 20% of water samples that were obtained in the Zoynan regions after 2012 appeared to contain *B. pseudomallei*-specific amplicons [36]. In contrast, the content of *B. pseudomallei* in samples obtained from rice field water is reportedly as high as 60% in Thailand [7]. The occupational risk of melioidosis is correlated with a farmer’s exposure to contaminated soil through agricultural activity; approximately 86% of those diagnosed with melioidosis also lived near a river in Liao [1,51]. Spatial analysis revealed that, in northern Australia, melioidosis is particularly endemic in Townsville, north Queensland, where one set of melioidosis cases was clustered in the area of the old course of a major waterway [52]. In the Northern Territory of Australia, all of the *B. pseudomallei*-positive environmental sites (*n* = 104) were permanently waterlogged, or waterlogged during the wet season, or were irrigated [53]. Water serving as a transmission vehicle for *B. pseudomallei* has been suggested when melioidosis case clusters are associated with the water supply [54,55]. Ingestion of or contact with water contaminated with *B. pseudomallei* is probably the transmission mode. However, more importantly, Taiwanese individuals, especially the elderly or patients, drink boiled water or routinely avoid being in close proximity to rivers or waterlogged areas.

The *A. lenticulata* PT-14 strain was isolated from a well after a flood disaster caused by a typhoon and could become a host of the endosymbiont *Burkholderia* spp., as the 16S RNA genes of the bacterium were detected in the amoeba. *A. lenticulata* is widely distributed in aquatic and terrestrial environments and frequently contains various bacterial endosymbionts, such as *Mycobacterium *sp., *Legionella *sp., and *Burkholderia *sp. [56,57,58]. In an in vitro study, *A. lenticulata* PT-14 displayed endosymbiosis with *B. pseudomallei* vgh07 (ST58) and vgh16 (ST1001) (data not shown). The levels of *A. lenticulata* distributed in river or drinking water reservoirs differ according to season; specifically, certain 18S RNA genes of *A. lenticulata* are frequently detected during September to November [59,60], a period in which typhoon invasion usually occurs. Typhoons are an important environmental phenomenon that triggers the incidence of melioidosis case clusters and is associated with high detection rates of *B. pseudomallei*-specific amplicons in water [13,35,36]. The intracellular survival of bacteria in amoeba was not only evolutionally designed to protect the bacteria from the harsh conditions of the environment, but also to enable the bacteria to develop a virulence that allows intracellular growth in mammals [61]. Thus, endosymbiotic *B. pseudomallei*, if they exist, not only interfere with microbiological cultures using filtered water, likely from sampling in Taiwan, but also become an alternative transmission mode of melioidosis from water to humans.

## 7. Conclusions

As an emerging and fatally infectious disease, melioidosis occurs because of exposure to soil or water contaminated with *B. pseudomallei*. Over 50% (223/444) of melioidosis cases were successively clustered in the Er-Ren River Basin in 2005 and in the Zoynan region in 2008–2012, and cases were also sporadically distributed throughout the Siaogang-Fengshan region in Taiwan. Subcutaneous inoculation, ingestion, and inhalation of contaminated soil or water are the main routes of melioidosis transmission. In 2005, individuals in the Taiwanese population acquired melioidosis seemingly through exposure to contaminated soil that flowed downstream from an upstream river area. While the upstream area had soil from which *B. pseudomallei* was isolated at high rates, the downstream region had a high incidence of melioidosis and high sero-prevalence for anti-*B. pseudomallei* antibodies. In 2008–2012, in the Zoynan region, melioidosis-clustered cases increased after typhoon events. In a prospective study, aerosols that contained *B. pseudomallei*-specific DNA were well distributed in the densely populated districts, but were rarely found in their surrounding areas. The concentration of specific DNA in aerosols was correlated with the melioidosis incidence and northwesterly wind in this endemic area. The isolation rate in the superficial layers of the contaminated crop field in the northwest was correlated with the PCR positivity of aerosols collected from the southeastern district over a two-year period. Because a strong northwest wind originating from a counterclockwise cyclone occurred during the wet season with a typhoon, the number of melioidosis cases was increased in a southeast district when *B. pseudomallei* was isolated from the northwestern region. Thus, melioidosis is airborne and was transmitted from contaminated soils to aerosols and/or to humans in Zoynan regions from 2008 to 2012. *B. pseudomallei* vgh07 (a representative strain of ST58) and its similar strain, which has a high virulence, have been prevalent in human and environmental isolates; however, the vgh16 strain (a representative strain of ST1001) and similar strains, with low virulence, were isolated from 2003 to 2016. If the ST1001 strain shows long-term persistence in animals or the environment as reservoirs, it could develop an increased virulence and potentially cause another outbreak in Taiwan. Although airborne transmission from aerosols to humans can occur in Taiwan, *B. pseudomallei* has a very low isolation rate in aerosols. Ingesting or coming into contact with water contaminated with *B. pseudomallei* is the likeliest mode of melioidosis transmission. However, more importantly, drinking boiling water and routinely avoiding proximity to rivers or waterlogged areas are common practices among the Taiwanese population. Most of the patients with melioidosis denied having contact with contaminated soil or water. Alternatively, the aim of endosymbiont *B. pseudomallei* to protect itself in harsh environments and develop a virulence to allow its intracellular growth in mammalian cells is likely another mode of melioidosis dissemination in Taiwan.
